# Sheathing of the Endovaginal Ultrasound Probe: Is It Adequate?

**DOI:** 10.1155/S1064744993000092

**Published:** 1993

**Authors:** Ronald Jimenez, Patrick Duff

**Affiliations:** 1 Division of Maternal-Fetal Medicine University of Florida College of Medicine Gainesville FL USA; 2 Department of Obstetrics and Gynecology University of Florida College of Medicine P.O. Box 100294 Gainesville FL 32610-0294 USA

## Abstract

The purpose of this prospective investigation was to compare two methods for sheathing of the endovaginal ultrasound-probe. The study was conducted over a 7-month period in 1991–1992. In the first half of the investigation, latex examination gloves were used to sheath the endovaginal probe; during the second half of the investigation, latex condoms were used. Following the ultrasound examination, the probes were inspected for gross contamination by the ultrasonographer. The sheaths were then tested for perforations by filling them with water to twice their usual volume and observing for leaks. Fifty unused gloves and condoms were similarly tested to determine the prevalence of preexisting defects. One hundred twenty-eight gloves and 102 condoms from patients were tested. Four gloves (3.1%, 95% C.I. 1.6–4.6%) and seven condoms (6.9%, 95% C.I. 4.4–9.4%) had perforations (NS). When the probe was covered by a glove, one instance of visible contamination occurred (0.78%, 95% C.I. 0–1.6%) compared with eight instances when the probe was covered with a condom (7.8%, 95% C.I. 5.2–10.4%, *P* < .007). The prevalance of preexisting defects in the 50 unused gloves was 2%, which is not significantly different from the prevalence in used gloves. There were no defects in the 50 unused condoms compared with 7 in the used condoms (*P* = .057). Visible contamination of the endovaginal probe with blood or genital tract secretions is more likely when condoms are used as sheaths. However, even gloves provide imperfect coverage of the probe, illustrating the need for thorough decontamination of the endovaginal instrument after each use.


Infectious Diseases in Obstetrics and Gynecology 1:37–39 (1993)
© 1993 Wiley-Liss, Inc.

### Sheathing of the Endovaginal Ultrasound Probe: Is It Adequate?

**Ronald Jimenez and Patrick Duff**

*Division of Maternal-Fetal Medicine, University of Florida College of Medicine, Gainesville, FL*

### Abstract

**The purpose of this prospective investigation was to compare two methods for sheathing of the endovaginal ultrasound probe. The study was conducted over a 7-month period in 1991–1992. In the first half of the investigation, latex examination gloves were used to sheath the endovaginal probe; during the second half of the investigation, latex condoms were used. Following the ultrasound examination, the probes were inspected for gross contamination by the ultrasonographer. The sheaths were then tested for perforations by filling them with water to twice their usual volume and observing for leaks. Fifty unused gloves and condoms were similarly tested to determine the prevalence of preexisting defects. One hundred twenty-eight gloves and 102 condoms from patients were tested. Four gloves (3.1%, 95% C.I. 1.6–4.6%) and seven condoms (6.9%, 95% C.I. 4.4–9.4%) had perforations (NS). When the probe was covered by a glove, one instance of visible contamination occurred (0.78%, 95% C.I. 0–1.6%) compared with eight instances when the probe was covered with a condom (7.8%, 95% C.I. 5.2–10.4%, $P < .007$). The prevalance of preexisting defects in the 50 unused gloves was 2%, which is not significantly different from the prevalence in used gloves. There were no defects in the 50 unused condoms compared with 7 in the used condoms ($P = .057$). Visible contamination of the endovaginal probe with blood or genital tract secretions is more likely when condoms are used as sheaths. However, even gloves provide imperfect coverage of the probe, illustrating the need for thorough decontamination of the endovaginal instrument after each use.**
© 1993 Wiley-Liss, Inc.

Key words

Contamination of ultrasound equipment, infection control

In recent years the medical community and public have become justifiably concerned about the risk of transmitting and acquiring certain infectious diseases during medical procedures. Most investigations have focused on disease transmission as a result of "invasive" surgical procedures. The risk of other less traumatic manipulations, such as endovaginal ultrasound, has not been clearly defined.

The first objective of the present investigation was to determine the frequency of manufacturing defects in two devices commonly used to cover the endovaginal probe: latex gloves and condoms. The second objective was to determine the subsequent frequency of perforation of these devices when they were stretched over the endovaginal ultrasound probe. The third objective was to determine the frequency with which the probe becomes visibly or grossly contaminated by blood and/or genital tract secretions as a result of an endovaginal scan.

### MATERIALS AND METHODS

This prospective study was conducted from October 1, 1991 to April 30, 1992, at Shands Hospital, University of Florida. During the study period, all endovaginal ultrasound examinations were performed by one of three attending physicians and

**Address correspondence to Dr. Patrick Duff, Department of Obstetrics and Gynecology, University of Florida College of Medicine, P.O. Box 100294, Gainesville, FL 32610-0294.**

Received December 30, 1992

Accepted March 11, 1993

four senior residents. For the first half of the investigation, the endovaginal probe (5 mHz, General Electric Medical Systems, Milwaukee, WI) was covered with a latex examination glove (Aladan Corp., Dothan, AL). Ultrasound gel was applied to the probe prior to placement of the probe in the second or third glove finger. Gel also was applied to the outside of the covered probe prior to its insertion into the vagina. The tip of the probe measured 2 × 2 × 5 cm. The total length of the probe and handle was 21 cm. The diameter and length of the opened, but unstretched glove finger, were 2.5 cm and 8.5 cm, respectively.

Midway through the study, we became aware that physicans working in a separate facility in our department were using condoms rather than gloves to cover the endovaginal probe. Subsequent to this discovery, we decided to complete the investigation using a different probe cover and, thereby, provide comparative data regarding the adequacy of the different sheaths. Accordingly, for the last half of the investigation, the probe was covered with a latex condom (Trojans, Carter-Wallace, NY). Gel was applied as previously described. The outer diameter of the rim of the condom was 3.7 cm. The length of the opened but unstreteched condom was 5 cm. At the time of this study, a fastener was not available to secure the upper end of the condom to the shaft of the probe.

At the conclusion of the ultrasound examination, the sheaths were placed in plastic bags. A label was affixed to each bag, and the sonographer was asked to indicate whether the sheath had an obvious perforation and whether there were blood or vaginal secretions on the probe. The probe was then cleansed with a dilute solution of sodium hypochlorite.

The gloves and condoms were tested for perforation by filling them with water to approximately twice their ususal volume and examining them for leakage of water. Fifty unused gloves and 50 unused condoms were similarly tested to determine the frequency of preexisting perforations. All tests of patency were performed by one of the authors (R.J.).

Our results are initially reported with descriptive statistics. Fisher's exact probability test was used to compare observed differences in frequency of perforation. $P < .05$ was considered statistically significant. Ninety-five percent confidence intervals also are reported, when appropriate.

### RESULTS

Four of 128 used gloves (3.1%, 95% C.I. 1.6–4.6%) had perforations. Seven of 102 used condoms (6.9%, 95% C.I. 4.4–9.4%) had perforations. This difference is not statistically significant ($P = .16$).

When the probe was covered by a glove, one instance of visible contamination occurred (0.78%, 95% C.I. 0–1.6%). In this case, there was no perforation in the glove, and contamination of the shaft of the probe apparently resulted from leakage of fluid around the cuff of the glove because of slippage of the glove during the examination. When a condom was used to cover the probe, eight cases of obvious contamination occurred (7.8%, 95% C.I. 5.2–10.4%). In six of these cases, the visible contamination coincided with the site of perforation in the condom. In two additional cases, there was no perforation in the condom, and leakage occurred around the open end of the condom onto the shaft of the probe. In one other instance in which a small perforation was present in the condom, no visible contamination was evident. The observed difference in frequency of visible contamination was highly significant ($P < .007$). There was no clustering of perforations or contamination in specimens submitted by any single sonographer.

The prevalence of defects in unused gloves was 2%, which is not significantly different from observed prevalence in used gloves. There were no preexisting defects in unused condoms compared with 6.9% in used condoms ($P = .057$).

### DISCUSSION

In recent years, physicians have become increasingly concerned about the risk of acquiring an infectious disease as a result of an occupational injury.[1,2] While this issue is a valid concern, health care workers also must be aware of their potential role in transmitting infection from themselves to their patients or from one patient to another. Contaminated medical equipment certainly is a possible mechanism for horizontal transmission of infection such as viral hepatitis, HIV infection, gonorrhea, chlamydia, and trichomoniasis.[3]

Although ultrasound imaging is not usually regarded as an invasive procedure, endovaginal scanning may pose some risk of infection to patients. In infected women, genital tract secretions may contain a high inoculum of infectious organisms. If the endovaginal ultrasound probe is not appropriately decontaminated after each procedure, and if breaks in the sheath used to cover the probe occur, potentially virulent organisms may be inoculated into a previously uninfected patient. Once they are inoculated onto the mucosal membrane, microbes may then gain access to the systemic circulation. Previous investigations of surgical glove perforations have demonstrated that manufacturing defects in unused gloves may be present and that breaks in the glove may occur during surgical manipulation.[4–8] Similarly, while latex condoms are clearly more effective barriers for transmission of infection than condoms made of natural substances, they do not provide perfect protection against breakage and subsequent disease transmission.

Our investigation demonstrates that perforations occur in approximately 3% (95% C.I. 1.6–4.6%) of latex gloves and 7% (95% C.I. 4.4–9.4%) of latex condoms used to sheath the endovaginal probe. Visible contamination of the probe with blood or genital tract secretions is more likely to occur when condoms are used as a sheath (7.8% vs. 0.78%, $P < .007$). This effect appears to be the result of three factors: a slightly higher rate of perforations in condoms, larger perforations in condoms, and additional leakage of genital secretions around the open end of the condom. Condoms are shorter than gloves and are not as easy to maintain in taut application against the shaft of the endovaginal probe without the aid of a fastener.

We recognize that our findings may be applicable only to certain types of gloves and condoms and to probes with the specific configuration used in this study. We also acknowledge that our study design has the potential for bias because the comparative portion of the investigation was introduced belatedly, and patients were not randomly assigned to the different types of sheaths. Nevertheless, we did not approach the second half of the investigation with any preconceived notion about which sheath was superior. There were no changes in study personnel, and the reporting of end points was uniform. Moreover, all examinations for perforations were performed by a single observer using a well established methodology.

Therefore, until additional information is published, we recommend the use of latex gloves rather than condoms to sheathe the vaginal probe. Even when gloves are used, some breakage and contamination may occur. Accordingly, the probe should be cleansed with an appropriate disinfectant, such as a dilute hypochlorite solution, after each use.

### REFERENCES

1. Recommendations for preventing transmission of human immunodeficiency virus and hepatitis B virus to patients during exposure-prone invasive procedures. MMWR 40: 1–9, 1991.
2. Welch J, Tilzey AJ, Webster M, Noah ND, Baratvala JE. Hepatitis B infections after gynaecological surgery. Lancet 1:205–207, 1989.
3. Possible transmission of human immunodeficiency virus to a patient during an invasive dental procedure. MMWR 39:489–493, 1990.
4. Rhoton-Vlasak A, Duff P. Glove perforations and blood contact associated with manipulation of the fetal scalp electrode. Obstet Gynecol 81:224–226, 1993.
5. Bennett B, Duff P. The effect of double gloving on frequency of glove perforations. Obstet Gynecol 78:1019–1022, 1991.
6. Chapman S, Duff P. Frequency of glove perforations and subsequent blood contact in association with selected obstetric surgical procedures. Am J Obstet Gynecol (in press).
7. Dodds RD, Guy PJ, Peacock AM, Duffy SR, Barker SGE, Thomas MH. Surgical glove perforation. Br J Surg 75:968, 1988.
8. Dodds RD, Barker SGE, Morgan NH, Donaldson DR, Thomas MH. Self-protection in surgery: The use of double gloves. Br J Surg 77:219–220, 1990.
